# Effects of fecal microbiota transplant on DNA methylation in subjects with metabolic syndrome

**DOI:** 10.1080/19490976.2021.1993513

**Published:** 2021-11-07

**Authors:** Eduard W. J. van der Vossen, Diogo Bastos, Daniela Stols-Gonçalves, Marcus C. de Goffau, Mark Davids, Joao P. B. Pereira, Andrew Y. F. Li Yim, Peter Henneman, Mihai G. Netea, Willem M. de Vos, Wouter de Jonge, Albert K. Groen, Max Nieuwdorp, Evgeni Levin

**Affiliations:** a Department of Vascular Medicine, Amsterdam University Medical Center, University of Amsterdam, Amsterdam, The Netherlands; b Horaizon BV, Delft, The Netherlands; c Wellcome Sanger Institute, Cambridge, UK; d Department of Genome Diagnostics, Amsterdam University Medical Center, University of Amsterdam, Amsterdam, The Netherlands; e Department of Experimental Internal Medicine, Radboud University, Nijmegen, The Netherlands; f Department for Genomics & Immunoregulation, Life and Medical Sciences Institute (Limes), University of Bonn, Bonn, Germany; g Laboratory of Microbiology, Wageningen University, Wageningen, The Netherlands; h Human Microbiome Research Program, Faculty of Medicine, University of Helsinki, Helsinki, Finland; i Tytgat Institute for Liver and Intestinal Research, Amsterdam University Medical Center, University of Amsterdam, Amsterdam, The Netherlands

**Keywords:** Gut microbiome, metabolome, FMT, epigenetics, machine learning

## Abstract

Accumulating evidence shows that microbes with their theater of activity residing within the human intestinal tract (i.e., the gut microbiome) influence host metabolism. Some of the strongest results come from recent fecal microbial transplant (FMT) studies that relate changes in intestinal microbiota to various markers of metabolism as well as the pathophysiology of insulin resistance. Despite these developments, there is still a limited understanding of the multitude of effects associated with FMT on the general physiology of the host, beyond changes in gut microbiome composition. We examined the effect of either allogenic (lean donor) or autologous FMTs on the gut microbiome, plasma metabolome, and epigenomic (DNA methylation) reprogramming in peripheral blood mononuclear cells in individuals with metabolic syndrome measured at baseline (pre-FMT) and after 6 weeks (post-FMT). Insulin sensitivity was determined with a stable isotope-based 2 step hyperinsulinemic clamp and multivariate machine learning methodology was used to uncover discriminative microbes, metabolites, and DNA methylation loci. A larger gut microbiota shift was associated with an allogenic than with autologous FMT. Furthemore, the data results of the the allogenic FMT group data indicates that the introduction of new species can potentially modulate the plasma metabolome and (as a result) the epigenome. Most notably, the introduction of *Prevotella* ASVs directly correlated with methylation of AFAP1, a gene involved in mitochondrial function, insulin sensitivity, and peripheral insulin resistance (Rd, rate of glucose disappearance). FMT was found to have notable effects on the gut microbiome but also on the host plasma metabolome and the epigenome of immune cells providing new avenues of inquiry in the context of metabolic syndrome treatment for the manipulation of host physiology to achieve improved insulin sensitivity.

## Introduction

Shifts in diet and lifestyle have been accompanied by an increase in the prevalence of Metabolic Syndrome (MetSyn) in Western societies.^1–3^ Although the underpinning pathophysiological mechanism driving MetSyn is not fully understood, an altered gut microbiome composition has been associated with obesity, glucose metabolism, and insulin sensitivity.^4–6^ Potential causality from altered microbiota composition to subsequent insulin resistance was derived from fecal microbiota transplant (FMT) studies both in animals^4,7,8^ and, more recently, in human trials.^9–11^ It is understood that cross-talk between the gut microbiome and host physiology occurs via the innate and adaptive immune system and/or via (dietary or mucus-derived) plasma metabolites. In this regard, the epigenomic modification of human genes by environmental factors (e.g., differential methylation of gene promoters involved in lipid metabolism and obesity) has been specifically linked to intestinal microbiota composition.^12–17^ While the mechanisms driving epigenetic changes in MetSyn are still unclear, recent studies underscore the potential role of microbial metabolites (notably short-chain fatty acids – SCFAs – such as propionate and butyrate) serving as co-substrates for epigenome-modifying enzymes.^18^ The increased insulin sensitivity in obese subjects with MetSyn upon receiving FMT from lean donors (allogenic FMT) was accompanied with changes in microbiota composition and intestinally-produced plasma metabolites.^9^

Following the clinical setup based on our randomized controlled study,^9^ we aimed to explore relationships between changes in intestinal microbiota, plasma metabolites, and DNA methylation in peripheral blood mononuclear cells (PBMCs) before and 6 weeks after allogenic or autologous FMT using a machine learning approach. Here we present an integrated view of the microbial, metabolic, and epigenetic data as well as biomarker signatures. The focus will be on the most relevant gut microbial ASVs affecting host metabolism and on notable epigenetic signatures associated with insulin resistance in subjects with MetSyn.

## Results

As described in detail previously,^9^ subjects with metabolic syndrome were treated with either autologous or allogenic FMT. Feces, fasting peripheral plasma and PBMCs were collected at baseline (pre-FMT) and after 6 weeks (post-FMT). These samples were used to obtain the gut microbiome composition, plasma metabolites, and host PBMC DNA methylation patterns in a subset of patients. The number of patients varied slightly per panel due to logistical reasons. A distinction was made between FMT responders and non-responders based on the peripheral insulin sensitivity, expressed as the rate of glucose disappearance (Rd).^10^ As previously reported, the response group was defined by an increase in Rd ≥10%.^9^

Data was available on 33 subjects for microbial analyses, comprising of 9 patients treated with autologous FMT and 24 with allogenic FMT. Of these 24 who were treated with an allogenic FMT, 11 were responders and 13 were non-responders. The plasma metabolites panel consisted out of 37 subjects of which 11 were treated with an autologous FMT and 26 subjects with allogenic FMT (13 responders and 13 non-responders). The epigenetic panel consisted of 20 subjects in which 7 subjects had been treated with an autologous FMT and 13 subjects with an allogenic FMT. Within the allogenic FMT group 7 subjects were classified as responders and 6 were non-responders. The demographics and clinical characteristics of the subjects within each of the three data modalities (microbes, metabolites, and epigenetics) are summarized in Table 1. For the responders and non-responders of the allogenic FMT group, the demographics are summarized in Supplementary Table S1. Percentagewise, the autologous group had lower numbers of responders (27%) than the allogenic treatment group (50%).

**Table 1.** Subject characteristics in the microbe-, metabolite-, and epigenetics panels

	Microbe panel (n = 33)				Metabolite panel (n = 37)				Epigenetics panel (n = 20)			
	Autologous (n = 9)		Allogenic (n = 24)		Autologous (n = 11)		Allogenic (n = 26)		Autologous (n = 7)		Allogenic (n = 13)	
	Responders (n = 2)		Responders (n = 11)		Responders (n = 3)		Responders (n = 13)		Responders (n = 2)		Responders (n = 7)	
	Baseline	6 weeks post-FMT	Baseline	6 weeks post-FMT	Baseline	6 weeks post-FMT	Baseline	6 weeks post-FMT	Baseline	6 weeks post-FMT	Baseline	6 weeks post-FMT
Age (yr)	54.4 ± 7.5		54.3 ± 6.8		55.0 ± 6.9		54.7 ± 6.8		56.2 ± 6.9		54.0 ± 7.2	
BMI (kg/m^2^)	36.2 ± 3.8	36.3 ± 4.3	34.4 ± 2.8	34.4 ± 2.6	35.9 ± 3.0	36.0 ± 4.1	34.3 ± 2.7	34.2 ± 2.7	36.8 ± 4.2	37.0 ± 4.7	32.9 ± 1.8	33.0 ± 1.7
SBP (mmHg)	156 ± 20		141 ± 16		153 ± 19		143 ± 16		157 ± 16		142 ± 18	
DBP (mmhg)	97 ± 14		87 ± 10		95 ± 13		88 ± 10		97 ± 10		87 ± 10	
HR (bpm)	72 ± 13		64 ± 8		73 ± 11*		64 ± 8*		71 ± 15		63 ± 6	
Cholesterol (mmol/L)	5.5 ± .9	5.3 ± 1.0	5.7 ± .9	5.6 ± 1.0	5.6 ± 1.0	5.4 ± .9	5.7 ± .9	5.6 ± 1.0	5.6 ± .9	5.5 ± 1.0	5.9 ± .9	5.9 ± .9
HDLc (mmol/L)	1.1 ± .2	1.0 ± .2	1.2 ± .2	1.1 ± .2	1.1 ± .2	1.0 ± .2	1.2 ± .2#	1.1 ± .2#	1.2 ± .2#	1.1 ± .3#	1.2 ± .2	1.1 ± .2
LDLc (mmol/L)	3.6 ± .7	3.6 ± .8	3.8 ± .8	3.9 ± .9	3.6 ± .6	3.7 ± .8	3.8 ± .8	3.8 ± .9	3.6 ± .6	3.7 ± .8	4.0 ± .7	4.1 ± .8
TG (mmol/L)	1.8 ± .6	1.6 ± .6	1.6 ± .9	1.4 ± .6	1.9 ± .8	1.6 ± .5	1.5 ± .9	1.4 ± .6	1.8 ± .7	1.6 ± .7	1.7 ± .9	1.6 ± .6
FFA (mmol/L)	.7 ± .1*	.6 ± .1*	.5 ± .2*	.5 ± .2*	.7 ± .1*	.7 ± .1*	.5 ± .2*	.5 ± .2*	.6 ± .1	.6 ± .1	.5 ± .2	.5 ± .2
REE (kcal/day)	2038 ± 239	2050 ± 295	1962 ± 182	1933 ± 173	2022 ± 231	2035 ± 267	1952 ± 187	1922 ± 173	2002 ± 253	2036 ± 335	1910 ± 180	1879 ± 118
Caloric intake (kcal/day)	2274 ± 434	2294 ± 263	2065 ± 465	2081 ± 527	2223 ± 411	2219 ± 289	2037 ± 466	2046 ± 526	2257 ± 486	2335 ± 246	2167 ± 415	2105 ± 441

Values are expressed as means ± SD. SBP: Systolic blood pressure; DBP: Diastolic blood pressure; HR: Heart rate; HDLc: High-Density Lipoprotein cholesterol; LDLc: Low-Density Lipoprotein cholesterol; TG: triglycerides; FFA: free fatty acids; REE: resting energy expenditure. Based on the Shapiro-Wilk test, either a parametric or non-parametric test was applied. For the difference between baseline and baseline (pre-FMT) and 6 weeks post-FMT, either the paired t-test or Wilcoxon signed-rank test was used (#p < .05). For the differences between autologous- and allogenic FMT, either the unpaired t-test or Mann-Whitney test was used (*p < .05).

Changes in three omics panels were analyzed using kernel-based methods^19,20^ that construct optimal separation hyperplanes between allogenic and autologous FMT subjects based on microbial, metabolite and epigenetic markers. The model was able to accurately discriminate allogenic from autologous FMT treated metabolic syndrome subjects based on the 16S rRNA amplicon analysis (test AUC = .82, permutations test *p*-value = .031, Supplementary Figure S1-2), targeted metabolomics (test AUC = .82, permutations test *p*-value = .006, Supplementary Figure S3-4) and DNA methylation profiles (test AUC = .78, permutations test *p*-value = .015, Supplementary Figure S5-6). The most discriminative markers from each of the three omics are highlighted in the following section. For the microbes panel, additional univariate analysis was done including the comparison between responders and non-responders. The last section includes an in-depth analysis of genes corresponding to the important DNA methylation loci found in the model.

### Changes in gut microbiome composition, plasma metabolites, and DNA methylation

**Figure 1.**
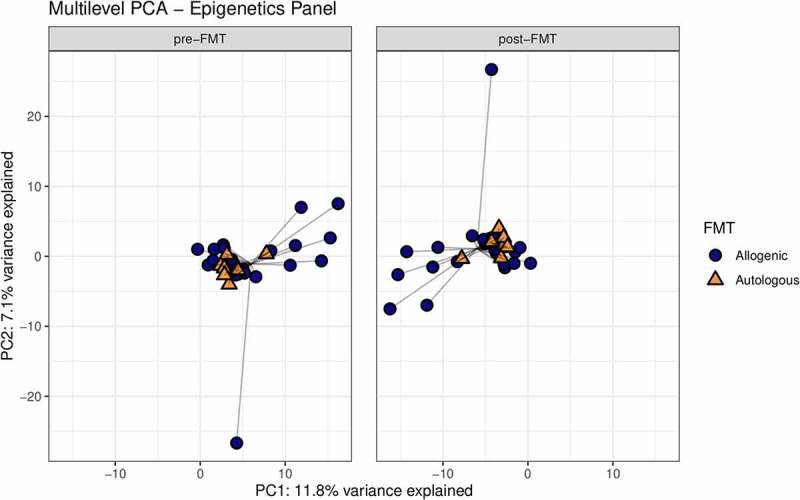
Multilevel PCA analysis plot displaying the differences of the gut microbial composition between the allogenic (blue) and autologous (Orange) groups before- and after FMT. The distance of each dot from the origin represents the amount of variation explained by the specific principal component. Note the mirroring in the plot pre- and post-FMT is due to within-subject deviation matrix in which two time points were used (pre-FMT and 6 weeks post-FMT) depicting the change over time. FMT was shown to have a significant effect independent of the groups (ADONIS2 R^2^ = .02082, *p*-value = .005, corrected for subject bias by permuting time within-subjects and treatment among subjects)

**Figure 2.**
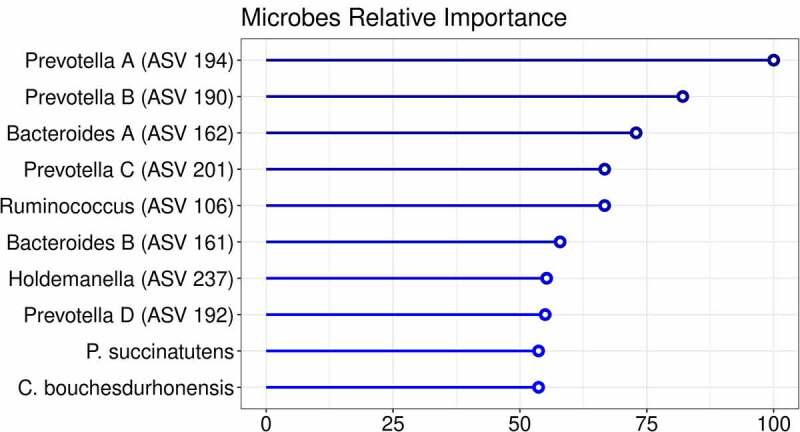
Importance plot showing the significant associations in the microbial panel that differentiate between changes upon autologous FMT versus changes upon allogenic FMT. The y-axis represents the top 10 most predictive microbial markers. The x-axis shows the relative importance of these microbial markers based on the permutation importance measure normalized between 0 to 100%. The color represents the largest change upon either autologous FMT (red) or allogenic FMT (blue)

**Figure 3.**
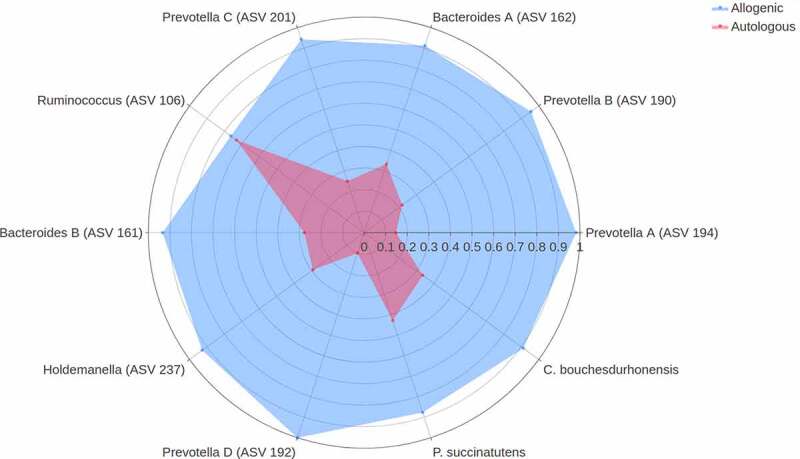
Spider plot depicting a panel of microbes that significantly differentiate between changes upon autologous FMT (red) versus changes upon allogenic FMT (blue). The axis of the spider plot represents the mean scaled changes for the top 10 most discriminative microbial markers. The microbial markers are based on 16s rRNA gene sequencing. Note that more ASVs belonging to the Prevotella and Bacteroides genus were identified and an alphabetical letter was added for the distinction between these ASVs

First, we studied the changes in gut microbiota composition between baseline and 6 weeks after autologous and allogenic FMT. There was a clear effect of FMT on the gut microbiota composition upon both allogenic FMT and autologous FMT as shown in the multilevel-PCA (Figure 1; PERMANOVA based on Euclidean distance after CLR transformation: R^2^ = .02082, *p*-value = .005).^21^ As expected, the Euclidian distance before and after FMT was significantly larger in the allogenic group (*p*-value = .004). Furthermore, we also compared the responders to the non-responders within both the autologous- and allogenic FMT groups and observed no large global differences (Supplementary figure S7). To pinpoint the most significant differences between the two FMT groups, changes (before and 6 weeks after FMT) in the three -omics panels were investigated. The top 10 most discriminative ASVs for separating autologous and allogenic FMT subjects are shown in Figure 2. We note that large shifts in the *Bacteroides stercoris* (ASV 161 and 162) and *Prevotella copri* (ASV 190, 192, 194, and 201) abundances were mainly confined to the allogenic FMT group whilst only *Ruminococcus bromii* (ASV 106) showed a large change in the autologous FMT group (Figure 3). The species names were included based on an additional BLAST search (see Supplementary Table S2 for the ASV sequences). To further elucidate potentially relevant changes pre- and post-FMT, we also investigated changes in the top 10 most discriminative ASVs univariately between the autologous and allogenic groups (Supplementary Figure S8), as well as between the responders and non-responders (Supplementary Figure S9). All of the ASVs found in the multivariate machine learning analysis show either borderline significant (.05 > FDR-corrected *p*-value > .1) or significant changes (FDR-corrected *p*-value ≤ .05) in the allogenic FMT group. Lastly, we directly correlated the percentage of *Prevotella* ASVs after treatment with the insulin levels and found a negative correlation (rho = −.38, *p*-value = .029).

**Figure 4.**
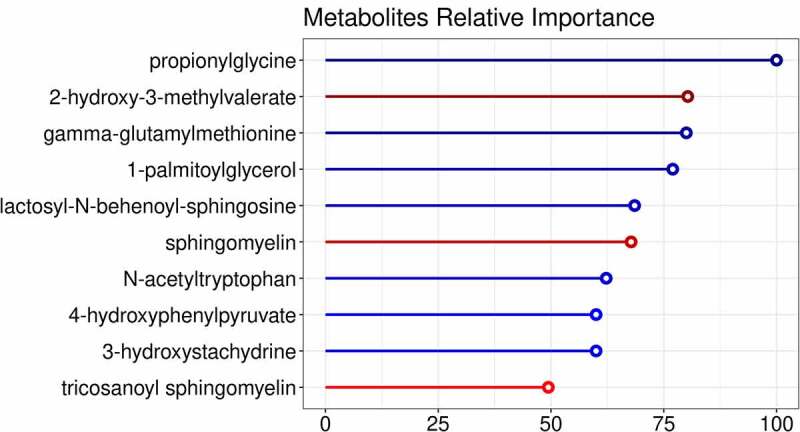
Importance plot showing the significant associations in the metabolic panel that differentiate between changes upon autologous FMT versus changes upon allogenic FMT. The y-axis represents the top 10 most predictive metabolic markers. The x-axis shows the relative importance of these microbial markers based on the permutation importance measure normalized between 0 to 100%. The color represents the largest change upon either autologous FMT (red) or allogenic FMT (blue)

**Figure 5.**
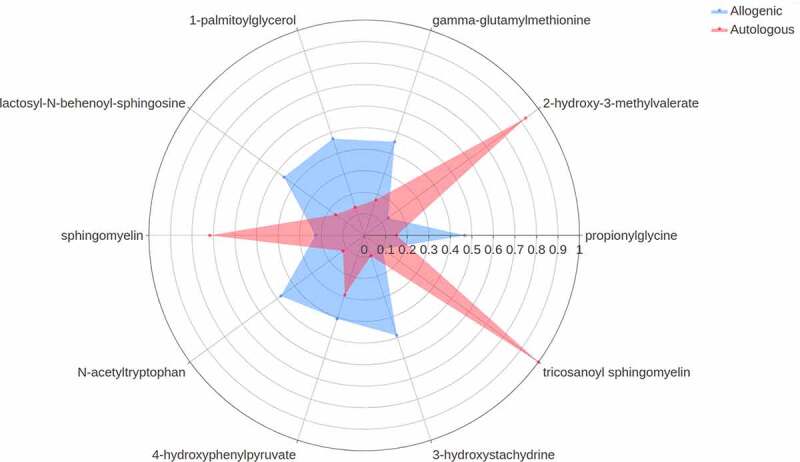
Spider plot depicting a panel of plasma metabolites that significantly differentiate between changes upon autologous FMT (red) versus changes upon allogenic FMT (blue). The axis of the spider plot represents the mean scaled changes for the top 10 most discriminative metabolic markers

Next, we studied changes in plasma metabolites. A significant effect of FMT on the plasma metabolite composition upon both allogenic FMT and autologous FMT was observed (PERMANOVA based on Euclidean distance after CLR transformation: R^2^ = .06701, *p*-value = .005). The model was able to distinguish between the two treatment groups. The top 10 most discriminative metabolites found in this model are shown in Figure 4. Notable changes in plasma metabolite levels were observed for both allogenic and autologous groups. In particular, in the allogenic FMT group metabolites such as lactosyl-N-behenoyl-sphingosine, 1-palmitoylglycerol, gamma-glutamylmethionine, propionylglycine, 3-hydroxystachydrine, N-acetyltryptophan, 4-hydroxyphenylpyruvate were increased (Figure 5). On the other hand, the metabolites described above were downregulated in the autologous FMT group with only tricosanoyl-sphingomyelin, 2-hydroxy-3-methylvalerate and sphingomyelin showing notable increases (Figure 5). Furthermore, we directly correlated the top 10 metabolites with the insulin levels post-FMT and found significant correlations with 4-hydroxyphenylpyruvate (rho = .442, *p*-value = .0062), Gamma-glutamylmethionine (rho = .36, *p*-value = .028), and propionylglycine (rho = −.345, *p*-value = .006). Univariate analyses were also used to study changes in plasma metabolites between the autologous and allogenic groups (Supplementary Figure S10) as well as between the responders and non-responders (Supplementary Figure S11). Within the allogenic group, 1-palmitoylglycerol and gamma-glutamylmethionine were observed to be significantly changed (FDR-corrected *p*-value ≤ .05).

**Figure 6.**
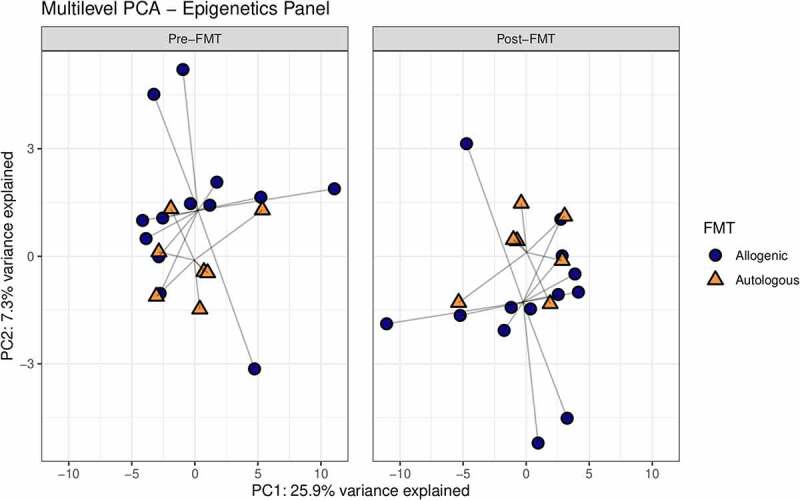
Multilevel PCA analysis plot displaying the differences of the DNA methylation of PBMCs signatures between the allogenic (blue) and autologous (Orange) groups before- and after FMT. The distance of each dot from the origin represents the amount of variation explained by the specific principal component. Note the mirroring in the plot pre- and post-FMT is due to within-subject deviation matrix in which two time points were used (pre-FMT and 6 weeks post-FMT)

**Figure 7.**
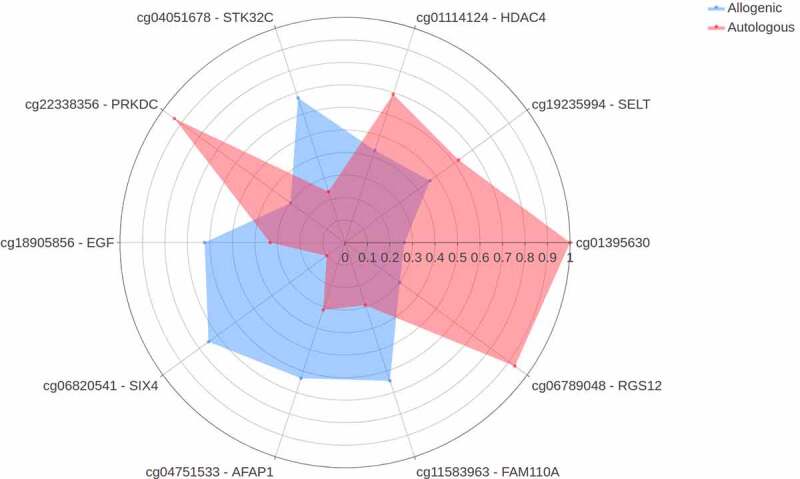
Spider plot depicting a panel of DNA methylation loci that significantly differentiate between changes upon autologous FMT (red) versus changes upon allogenic FMT (blue). The axis of the spider plot represents the mean scaled changes for the top 10 most discriminative loci

**Figure 8.**
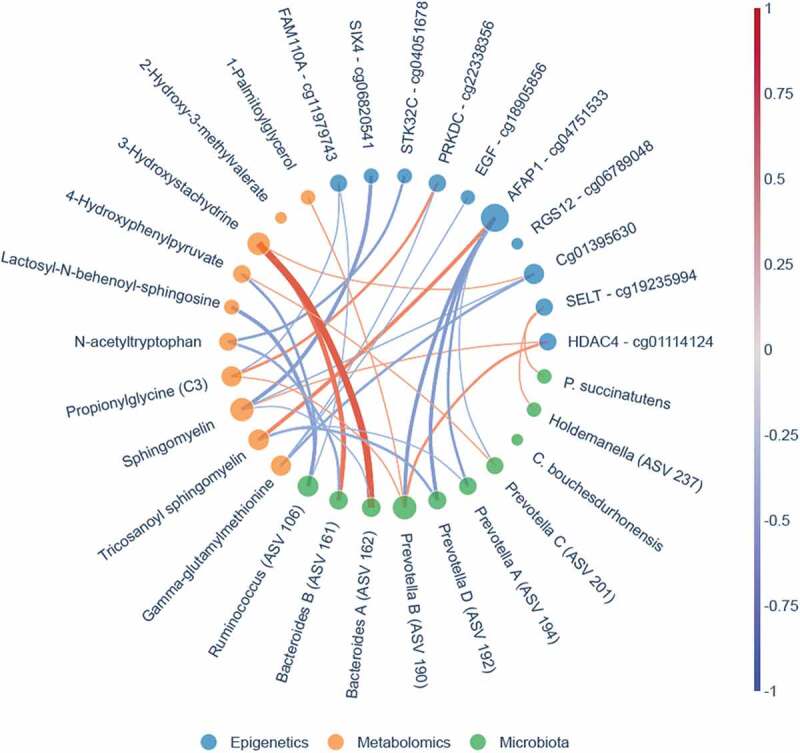
Bokeh network graph on multi-omics showing correlations between the three different panels. The top 10 most discriminative predictive markers of the microbial panel (green nodes), metabolite panel (Orange nodes) and epigenetics panel (blue nodes) are displayed. Lines between the different nodes represent Spearman correlations. A red line represents a strong positive correlation, whereas the blue line represents a strong inverse correlation. The thickness of the line represents the degree of the correlation. The size of the node is dependent on the number of correlations where more correlations lead to larger nodes. The microbial markers are based on 16s rRNA gene sequencing. Note that more ASVs belonging to the *Prevotella* and *Bacteroides* genus were identified and an alphabetical letter was added for the distinction between these ASVs

**Figure 9.**
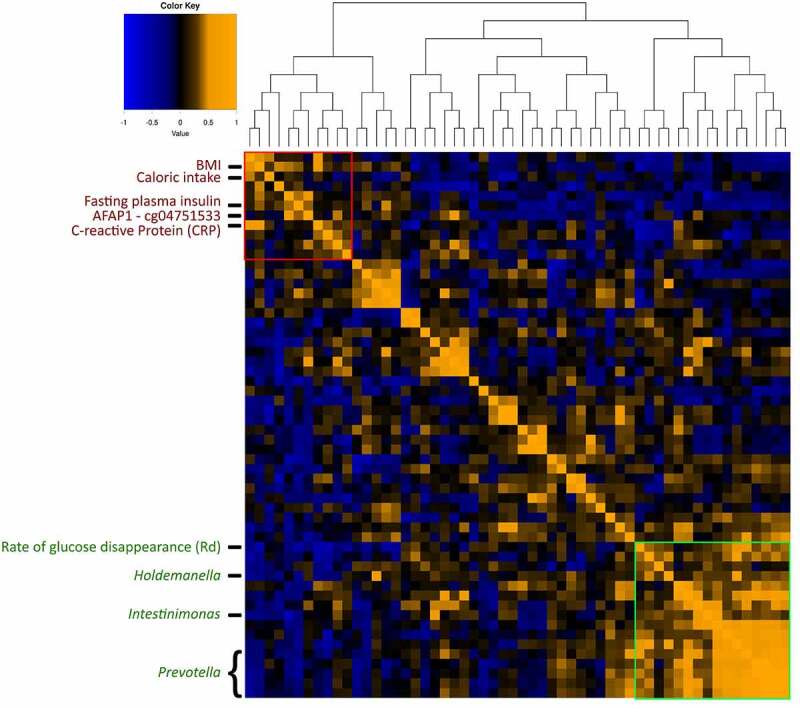
Heatmap including all correlations between and within the three different panels and clinical parameters post-FMT (6-weeks after intervention). The distance matrix was created using Euclidean distance. Hierarchical clustering was done using the complete agglomeration method. Strong positive correlations are depicted by the Orange color, strong negative correlations are depicted by the blue color. Different blocks are highlighted. The red block depicts unhealthy variables that strongly correlate with each other. The green block represents the strong correlation between different ASVs of *Prevotella* and *Intestinimonas* and its positive correlation with the rate of glucose disappearance

Hereafter, we set out to identify differences in change in DNA methylation upon either autologous or allogenic FMT. The multilevel PCA constructed on data showed separation between the two treatment groups (Figure 6). When comparing the responders and non-responders within both the autologous- and allogenic FMT groups, no large differences were observed (Supplementary Figure S12). The mean scaled differences for the top 10 most discriminative DNA methylation loci in host PBMCs are shown in Figure 7. We observed a larger shift in DNA methylation in the allogenic group for the gene Actin filament-associated protein 1 (AFAP1; cg04751533), which was larger in the responders of the allogenic group (Supplementary Figure S13). In addition, larger shifts in DNA methylation in the allogenic group were observed in SIX homeobox 4 (SIX4; cg06820541), Epidermal growth factor (EGF; cg18905856), Serine/Threonine Kinase 32 C (STK32C; cg04051678), and protein FAM110A (cg11583963). In the autologous group larger changes in methylation were observed for Histone deacetylase 4 (HDAC4; cg01114124), Protein kinase, DNA-activated, catalytic subunit (PRKDC; cg22338356), Selenoprotein T (SELT; cg19235994), and Regulator Of G-Protein Signaling 12 (RGS12; cg06789048), as shown in Figure 7. Furthermore, we directly correlated the top 10 DNA methylation loci with insulin levels and a trend with methylation of cg04751533 of AFAP1 (rho = .384, *p*-value = .095). Additional mixed-design ANOVA tests were applied to test if the proportions of PBMCs cell fractions (CD4, CD8, NK, B-cells, and monocytes) were confounders yet this was not found to be the case (Supplementary Table S3; Supplementary Figure S14). After identifying the most discriminative markers for each of the -omics panels, we studied multi-omics interactions upon FMT by combining all parameters mentioned above. A correlation matrix was constructed containing changes in the gut microbes, the changes in DNA methylation as well as the changes in plasma metabolites (Spearman rho, Supplementary Table S4) to examine the interactions between differential changes caused by autologous/allogenic FMT (see Figure 8). In the combined data of both the allogenic and autologous groups, there were 4 significant correlations between different ASVs of *Prevotella* associated with the methylation of AFAP1 gene (rho = −.471, −.565, −.584, and −.592, with the respective *p*-values of .036, .0094, .0069, and .006) and 2 correlations between different species of *Bacteroides* with the 3-hydroxystachydrine plasma metabolite (rho = .658, and .751, with the respective *p*-values of .0016, and .0001). When examining links between the changes in plasma metabolite levels and DNA methylation loci, sphingomyelin had the most correlations with DNA methylation loci, both positive (HDAC4, rho = +.474, *p*-value = .0349) and negative (Sine oculis homeobox homolog 4 – SIX4; rho = −.57, *p*-value = .0081). Besides the correlation matrix between the top 10 most discriminative biomarkers from each panel that distinguish the autologous from allogenic FMT group, a correlation matrix between important microbes, metabolites, DNA methylation loci, and clinical parameters was constructed based on post-FMT (6 weeks) measurements (Figure 9; Supplementary figure S15). Clear positive correlations were observed between different ASVs of *Prevotella, Intestinimonas* and *Holdemanella*, with the rate of glucose disappearance (Rd) and a negative correlation was observed between this block of variables and fasting plasma insulin. Furthermore, though upregulated in the allogenic group, a negative correlation between *Bacteroides* and *Prevotella* ASVs was observed.

**Figure 10.**
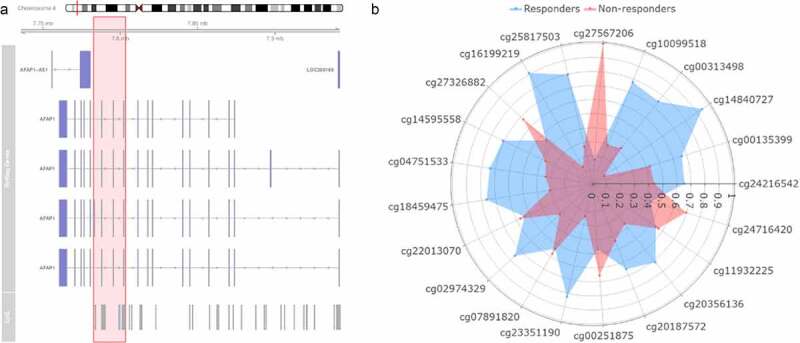
Exploration of neighboring CpGs of cg04751533 found in the model. (a) Visual representation of the location on the chromosome, the genes located on this place and the neighboring CpGs that are present in the Infinium methyl 450k array. The part highlighted in red includes the neighboring CpGs investigated. (b) Spider plot depicting the neighboring CpGs of cg04751533 that differentiate between changes upon the non-responders (red) versus changes upon the responders (blue)

A more in-depth analysis was done to further elucidate the possible clinical relevance of epigenetic biomarkers that had multiple correlations with markers from the microbial and metabolite panels. Changes in DNA methylation loci found in the gene AFAP1 (cg04751533) was investigated by verifying whether neighboring CpGs were displaying similar patterns. Indeed, differences in the methylation patterns for the responder- and the non-responder group receiving allogenic FMT for AFAP1 were observed (Figure 10; Supplementary figure S16). Neighboring CpGs of cg04751533 in the AFAP1 gene showed a larger decrease in methylation in the responders.

## Discussion

The main aim is to study the reciprocal relationship between gut microbiota changes and changes in the plasma metabolic profile as well as changes in DNA methylation patterns in PMBCs as induced by FMT and relate this to insulin resistance as determined by the gold standard 2-step hyper insulinemic stable isotope-based clamp in MetSyn subjects. Three machine learning models, one for each of the three datasets (microbial, metabolic and epigenetic), were deployed to accurately differentiate between allogenic and autologous FMT receivers and to extract the most discriminative biological features from each biological dimension. Changes in the gut microbiota composition were dependent on and discriminative of the FMT treatment group, with a higher response variation in the allogenic group than in the autologous group as previously described.^9–11^ This may be explained by complex interactions between the existing microbiota and the allogenic donor microbiota, with the colonization of allogenic microbes, dependent on the composition of the existing host microbiome. Interestingly, the pattern of microbial change post-FMT showed that a small subset of the top 10 most discriminative ASVs markedly increased in the allogenic but not in the autologous FMT group.

### Prevotella-Bacteroides interplay in FMT in relation to SCFA production

The most discriminative ASVs stemming from our analysis are *Prevotella* and *Bacteroides*. Investigating post-FMT composition, a clear negative correlation between *Bacteroides* ASVs and *Prevotella* ASVs is observed. Previous literature states that *Prevotella* has a protective function against *Bacteroides*-induced glucose intolerance and that both these bacteria tend to compete for the same niche.^22^ Both *Bacteroides* and *Prevotella* are known producers of the SCFAs acetate, propionate and may contribute to butyrate production via cross-feeding interactions with butyrate producers.^23,24^ These SCFAs have been linked to improved glucose metabolism. *Prevotella* dominant gut microbiome compositions are however known to be able to achieve higher SCFA production levels than *Bacteroides* dominated gut microbiome compositions.^23^ No butyrate producers were however present within the bacterial top 10. Investigation outside the top 10 most discriminative markers led to an ASV of the *Intestinimonas* genus, which clusters together with the *Prevotella* ASVs (Figure 9). The *Intestinimonas* genus is known to produce mainly butyrate not only from sugars but also from acetate and lactate, as well as lysin and fructose lysin.^25^ Besides being used by colonocytes as a primary energy source and by the liver for lipogenesis, butyrate is a powerful inhibitor of histone deacetylase which regulates gene expression.^26,27^ Animal and *in vitro* studies have generally found a beneficial effect of butyrate and acetate on glucose homeostasis and insulin sensitivity.^28^

### Alteration in plasma metabolites related to lipid metabolism

The model for the metabolite panel showed a small subset of 10 plasma metabolites which changed upon FMT. Interestingly, most of these plasma metabolites are involved in lipid metabolism. Many are sphingolipids and though not many significant changes were observed univariately, a clear distinction between *Bacteroidetes*-derived sphingolipids and host sphingolipids is of importance in regards to inflammation in the host.^29^ Furthermore, the actions of cellular bioactive sphingolipids are increasingly of interest due to their roles in inflammation and diabetes.^30^

Both sphingomyelin and tricosanoyl sphingomyelin were found to be less altered upon allogenic FMT group in comparison to the autologous FMT group. These metabolites are generated via sphingolipid metabolism. Reduced sphingolipid metabolism and subsequently the accumulation of these metabolites has been linked to insulin sensitivity in mice.^31^ Furthermore, lactosyl-N-behenoyl-sphingosine, a ceramide also belonging to the sphingolipids, is decreased in the responders of the allogenic FMT group. A decreased amount of ceramides, either via inhibition of synthesis or stimulation of degradation, has shown to improve insulin sensitivity.^32^

2-hydroxy-3-methylvalerate was shown to change more in the autologous group and increases mildly in the non-responder group of the autologous group. 2-hydroxy-3-methylvalerate has an effect on peroxisome proliferator-activated receptor-alpha, which is a major regulator of lipid metabolism in the liver. This in turn is related to reduced physical function in older adults.^33^ Furthermore, 2-hydroxy-3-methylvalerate was found to be significantly positively associated with insulin resistance and both intermuscular- and subcutaneous adipose tissue inflammation.^34^ Gamma-glutamylmethionine was also found to be a highly discriminative biomarker increased in the allogenic FMT group. The first step in degradation of glutathione produces this gamma-glutamylamino acid to transport amino acids in mammalian tissue. This increase in gamma-glutamylamino acids has been suggested to reflect decreased glutathione levels.^35^ It was shown that glutathione is decreased in older mice and when these levels are restored to the levels of glutathione found in young mice, improved glucose metabolism and reduced insulin resistance were observed.^36^ 1-palmitoylglycerol was found to be increased in the allogenic FMT group in comparison to the autologous FMT group. Within the allogenic group, it is border significantly increased in the non-responders and significantly increased in the responders. This monoacylglycerol has been shown to have a synergistic effect on insulin secretion when combined with GLP-1, Carbamylcholine or α-ketoisocaproate in β cell-specific α/β-Hydrolase domain-6-KO mice.^37^ Finally, propionylglycine has also shown to be an important biomarker. Plasma levels of propionyl glycine have been long associated with the early stage of diabetic kidney disease.^38^ Furthermore, it is a derivative of the SCFA propionate which has been linked to DNA methylation correcting for aberrant expressions of proteins.^39^

### Changes in DNA methylation loci upon FMT

One of the most discriminative loci was associated with the AFAP1 gene, a gene involved in mitochondrial function. Interestingly, all *Prevotella* ASVs which were found in the microbial analysis were inversely correlated with cg04751533 in AFAP1. cg04751533 was previously investigated and a decrease in methylation of this CpG led to an increase in diastolic blood pressure,^40^ Although no relation with insulin resistance has been reported before, increased AFAP1 expression was linked to altered glucose metabolism in human brain capillary endothelial and associated with inflammation.^41^

Moreover, it is interesting to note that propionylglycine shows a linear correlation with PRKDC, a gene that encodes a protein that acts as a molecular sensor for DNA damage.^42^ Furthermore, it was previously shown that the PRKDC gene was downregulated in the PBMCs of subjects with type-2 diabetes in comparison with healthy subjects.^43^

Finally, the relation between gut microbiota changes and methylation of the biomarker gene histone deacetylase HDAC is substantiated by several publications that show that this gene is implicated in mechanisms mediating the interaction between environmental factors and the genome, with particular importance in the pathogenesis of metabolic syndrome and type 2 diabetes.^44^ Large scale Genome-Wide Association Studies (GWAS) recently linked HDAC to insulin secretion.^45^ cg01395630 methylation was inversely correlated with plasma gamma-glutamylmethionine levels. Gamma-glutamylmethionine is a dipeptide, composed of gamma-glutamate and methionine and the latter has been linked to DNA methylation.^46^ However, relationships between methylation of individual loci and gene expression profiles are complex, with many loci lacking clear gene associations and further mechanistic studies are required to dissect these relationships.

The findings of our study should be considered in light of some limitations. Despite the fact that we obtained statistically significant results in this relatively small (paired) dataset, an increase in the number of subjects that undergo allogenic and autologous FMT may further improve the stability and reliability of the discovered biomarkers. In addition, we focused here on the fecal microbiota but previously showed that small intestinal signaling may also be relevant in the context of MetSyn.^10^ In addition, only PBMC signatures were used for the methylation panel and not the monocytes’ DNA, which might have shown a stronger and more specific signal. Lastly, to further strengthen the findings in this study, a functional investigation of the microbial community has yet to be done. This could confirm any causal effect between the microbial community and consequently the plasma metabolites and changes in DNA methylation.

In conclusion, we here present an integrated view of gut microbial, metabolic and host epigenetic data based on a randomized controlled trial that aimed to study the effect of FMT on metabolism in subjects with MetSyn. We found a marked effect of the FMT source (lean-donor or autologous) on the three data modalities and clinical parameters. This is the first time investigating the relations between microbes, plasma metabolites and epigenetics in a biomarkers-based fashion to our knowledge. This investigation supports a potential causal connection between the three panels in the context of MetSyn.

## Material and methods

### Clinical study inclusion/exclusion criteria and study design

Subjects were recruited and screened for characteristics of the metabolic syndrome [1]. In brief, we included treatment naïve adult (age 21–69 yrs.) men of European ancestry, who were obese (body mass index (BMI) ≥30 kg/m^2^), fulfilled the National Cholesterol Education Program (NCEP)-criteria for metabolic syndrome (≥3/5: fasting plasma glucose ≥5.6 mmol/L, triglycerides ≥1.7 mmol/L, waist-circumference >102 cm, high-density lipoprotein (HDL)-cholesterol <1.03 mmol/L, blood pressure ≥130/85 mmHg) and who were otherwise healthy. Exclusion criteria were a history of recent weight loss, cardiovascular event, cholecystectomy and the use of any medication known to influence gut microbial composition in the last three months (including proton pump inhibitors, antibiotics and pre-/pro-/synbiotics) or targeting metabolic diseases (e.g., lipid-lowering, anti-diabetic and/or anti-hypertensive drugs). Lean (BMI <25 kg/m^2^), omnivorous, healthy Caucasian males were also recruited to serve as fecal donors. They completed questionnaires regarding dietary and bowel habits, travel history, comorbidity including (family history of) diabetes mellitus and medication use. Donors were screened for the presence of infectious diseases as previously published (van Nood et al., 2013).^47^ Blood was screened for presence of (antibodies to) human immunodeficiency virus; human T-lymphotropic virus; Hepatitis A, B, and C; cytomegalovirus (CMV); Epstein–Barr virus (EBV); strongyloides; amoebiasis and lues. Presence of infection resulted in exclusion, although previous, non-active infections with EBV and CMV were allowed. Donors were also excluded if screening of their feces revealed the presence of pathogenic parasites (e.g., blastocystis hominis, dientamoeba fragilis, giardia lamblia), bacteria (*Shigella, Campylobacter, Yersinia, Salmonella*, enteropathogenic *E. coli* and *Clostridium difficile*) or viruses (noro-, rota-, astro-, adeno (40/41/52)-, entero-, parecho- and sapovirus) at AMC department of Clinical Microbiology and Virology. Written informed consent was obtained from all subjects. The study was approved by the local Institutional Review Board of the Academic Medical Center (AMC) in Amsterdam, the Netherlands, and conducted at the AMC in accordance with the Declaration of Helsinki. The study was registered at the Dutch Trial Register (number 2705). We performed a double-blind randomized controlled trial studying the effect of allogenic (lean donor) gut microbiota infusion 6, using autologous infusion as the control. Donors and recipients were randomly matched.^9^ Gut microbiome composition, fasting plasma metabolites (Metabolon) and PBMCs DNA methylation signatures were measured at baseline (pre-FMT) and after 6 weeks (post-FMT). For each -omics dataset, the number of subjects used is visualized in supplementary figure S17.

### Intestinal microbiota

DNA was extracted from fecal material using a repeated bead beating protocol⁠ (described in detail as “method 5” in Costea et al.).^48^ DNA was purified using Maxwell RSC Whole Blood DNA Kit. 16S rRNA gene amplicons were generated using a single step PCR protocol targeting the V3-V4 region^49^⁠. PCR products were purified using Ampure XP beads and purified products were pooled in equimolar fashion. The libraries were sequenced using a Illumina MiSeq platform using V3 chemistry with 2 × 250 cycles. Forward and reverse reads were truncated to 240 and 210 bases, respectively, and merged using USEARCH (v.11).^50^ The minimum percentage identity of alignment was set to 80%. Reads with an expected error rate higher than 2 and reads that were shorter than 380 bases were removed. Amplicon sequence variants (ASVs) were inferred for each sample individually with a minimum abundance of 4 reads using the UNOISE3 algorithm.^51^ Merged reads that passed screening were subsequently mapped against the set of inferred ASVs in order to construct a count table. Taxonomy was assigned to ASVs using the RDP classifier^52^ and the SILVA^53^ 16S ribosomal database (v.132). The count table was rarefied at 34124 reads. Paired microbiome profiles were obtained from 33 subjects (24 from the allogenic and 9 from the autologous group).

### Plasma metabolites

Global targeted metabolite profiling was performed at both timepoints on fasting peripheral plasma by Metabolon (Morrisville, NC, USA) using ultra high-performance liquid chromatography coupled to tandem mass spectrometry, as previously described (Koh et al; section ‘Metabolite analysis’).^54^ After obtaining data on the plasma metabolites, further processing was done. First, metabolites that were either all zero or constant, were omitted. Next, the values of each metabolite across all samples were rescaled to 1 in which missing values are imputed with the lowest present value for its respective metabolite. Hereafter, batches were combined. Paired plasma metabolites were obtained from 37 subjects (26 from the allogenic and 11 from the autologous group).

### Epigenetic measurements

Whole blood was obtained from the patients at both timepoints and the interphase layer of peripheral blood mononuclear cells (PBMCs) was extracted after centrifugation with Ficoll® Paque Plus cell separation media. The proportion of the different PBMC cell fractions was estimated based on global PBMC DNA methylation patterns.^55^ DNA was subsequently isolated using a QIAamp® DNA blood mini kit following the manufacturer’s blood spin protocol and bisulfite-treated using the Zymo EZ DNA methylation kit. Whole-genome DNA methylation profiles were quantified using the Illumina HumanMethylation450k BeadChip Array, at ServiceXS in Leiden, the Netherlands. The methylation data was imported into the R statistical programming environment (version 3.2.2) using the Bioconductor package minfi (version 1.16.0).^56^ Initial quality control was performed using the MethylAid package,^57^ whereby the quality of each sample was assessed using the internal control probes located on the BeadChip array. Probes known to be promiscuous,^58^ located on the sex chromosomes, or associated to CpGs with known SNPs (minor allele frequency >0) were removed. The remaining probes were normalized using the functional normalization method,^59^ after which percentage methylation (β-values) per locus were extracted for downstream analyses. Paired DNA-methylation profiles were obtained from 20 of the 38 subjects (13 from the allogenic and 7 from the autologous group).

### Machine learning analysis

Panels of gut microbial, metabolic and epigenetic biomarkers were identified in order to discriminate between allogenic and autologous subjects upon fecal microbial transplant (FMT). For each panel, all available data was included. This includes for 33 subjects for the microbiome panel (24 allogenic and 9 autologous), 37 subjects for the metabolite panel (26 allogenic and 11 autologous), and 20 subjects for the epigenome panel (13 allogenic and 7 autologous). The relative change of microbial abundances, metabolites and the absolute changes in the DNA methylation levels (measured as percentage methylation per locus) at baseline and 6 weeks post-FMT were computed. For each of the three datasets, a support vector machine model was determined to achieve the best performance. To avoid over-fitting, leave one out cross-validation^60^ over the training partition (80%) of the data was applied. The remaining data (20%) was used as the test dataset. A permutation (randomization test)^61^ was used to evaluate statistical validity of the results. In the permutation test, the outcome variable (i.e., allogenic or autologous FMT) was randomly reshuffled 1000 times while the corresponding -omics profiles were kept intact. In order to obtain the biomarker signatures, a permutation importance measure was used. Python v. 3.7 (www.python.org), with packages NumPy, SciPy and Scikit-learn were used for implementing the stacking model and R version 4.0.0 for the visualizations.

### Statistical analysis of the profiles

The statistical analysis on machine learning models was done using a permutation test in python 3.7. All other statistical analysis was performed using R version 4.0.0. Within-group changes were tested with the paired Wilcoxon-signed rank tests or paired t-test. Mann-Whitney U-test or the unpaired t-test were applied to compare independent groups. Either parametric or non-parametric tests were used, based on the result of the Shapiro-Wilk test for normality.

To test the effect of FMT on the microbiome, metabolites and epigenetics panel, a permutational multivariate analysis of variance using distance matrices were applied (ADONIS).^62^ A stratified permutation was done to correct for subject bias. Here, the time within a subject and treatment among subjects were permuted. The number of permutations used was 199.

For univariate analysis on the microbiome- and metabolite data, a false discovery rate corrected *p*-value below .05 was considered significant, corrected for multiple testing. For each cell fraction of PBMCs, a mixed-design ANOVA model was built using group (Allogenic/Autologous) and time (Baseline/6 weeks post-FMT) as fixed effects and Subject ID as a random effect. For correlation analysis between the different panels, Spearman’s rho was applied. The significance level was below .05.

## Supplementary Material

Supplemental Material

## Data Availability

Deidentified patient data on the gut microbiome, plasma metabolites and DNA methylation loci per subject will be publicly available following publication upon request.

## Abbreviations

ASVs – Amplicon sequence variants

AUC – Area Under Curve

FMT – Fecal Microbiota Transplant

HDAC – Histone deacetylase

MetSyn – Metabolic Syndrome

PBMCs – Peripheral Blood Mononuclear Cells

PCA – Principal Component Analysis

PRKDC – Protein kinase, DNA-activated, catalytic subunit

Rd – rate of glucose disappearance

RGS12 – Regulator Of G-Protein Signaling 12

ROC – Receiver Operating Characteristics

SCFAs – Short Chain Fatty Acids

SELT – Selenoprotein T

SIX4 – Sine oculis homeobox homolog 4
