# Supplementary material for: Effects of fecal microbiota transplant on DNA methylation in subjects with metabolic syndrome
Source: Gut Microbes. 2021 Nov 7;13(1):1993513. doi: 10.1080/19490976.2021.1993513 (PMC8583152; doi:10.1080/19490976.2021.1993513)
Supplement: Supplemental Material [file KGMI_A_1993513_SM2105.zip › Supplemental figure captions.docx]

# Supplemental Information - Figure Captions

**Figure S1-2.** ROC-AUC plot and the permutation significance test results for the microbial machine learning model.

**Figure S3-4.** ROC-AUC plot and the permutation significance test results for the plasma metabolites machine learning model.

**Figure S5-6.** ROC-AUC plot and the permutation significance test results for the epigenome machine learning model.

**Figure S7.** Multilevel PCA analysis plot displaying the differences of the gut microbial composition between the responders (triangle) and non-responders (circle) within the allogenic (blue) and autologous (orange) groups before- and after FMT. The distance of each dot from the origin represents the amount of variation explained by the specific principal component. Note the mirroring in the plot pre- and post-FMT is due to within-subject deviation matrix in which two time points were used (pre-FMT and 6 weeks post-FMT) depicting the change over time.

**Figure S8.** Boxplots of the top 10 microbes found in the machine learning model. Univariate analysis was done on the autologous and allogenic groups. Within-group changes were tested with the paired Wilcoxon-signed rank tests. Mann-Whitney U-test was used to compare independent groups. All p-values were corrected for false discovery rate. P-value. Significance codes are as following: p<0.001 (***), p<0.01 (**), p<0.05 (*), p<0.1 (.).

**Figure S9.** Boxplots of the top 10 microbes found in the machine learning model. Univariate analysis was done on the responder and non-responder groups receiving allogenic FMT. Within-group changes were tested with the paired Wilcoxon-signed rank tests. Mann-Whitney U-test was used to compare independent groups. All p-values were corrected for false discovery rate. P‑value. Significance codes are as following: p<0.001 (***), p<0.01 (**), p<0.05 (*), p<0.1 (.).

**Figure S10.** Boxplots of the top 10 plasma metabolites found in the machine learning model. Univariate analysis was done on the autologous and allogenic groups. Within-group changes were tested with the paired Wilcoxon-signed rank tests. Mann-Whitney U-test were used to compare independent groups. All p-values were corrected for false discovery rate. P-value. Significance codes are as following: p<0.001 (***), p<0.01 (**), p<0.05(*), p<0.1 (.).

**Figure S11.** Boxplots of the top 10 plasma metabolites found in the machine learning model. Univariate analysis was done on the responder and non-responder groups receiving allogenic FMT. Within-group changes were tested with the paired Wilcoxon-signed rank tests. Mann-Whitney U-test were used to compare independent groups. All p-values were corrected for false discovery rate. P-value. Significance codes are as following: p<0.001 (***), p<0.01 (**), p<0.05(*), p<0.1 (.).

**Figure S12.** Multilevel PCA analysis plot displaying the differences of the DNA methylation of PBMCs signatures between the responders (triangle) and non-responders (circle) within the allogenic (blue) and autologous (orange) groups before- and after FMT. The distance of each dot from the origin represents the amount of variation explained by the specific principal component. Note the mirroring in the plot pre- and post-FMT is due to within-subject deviation matrix in which two time points were used (pre-FMT and 6 weeks post‑FMT).

**Figure S13.** Boxplots of AFAP1 - cg04751533 comparing (A) pre to post-FMT in the autologous and allogenic FMT groups as well as (B) the responders and non-responders in the allogenic FMT group.

**Figure S14.** Boxplots for PBMC cell fractions.

**Figure S15.** Heatmap including all correlations between and within the three different panels and clinical parameters post-FMT (6-weeks after intervention). The distance matrix was created using Euclidean distance. Hierarchical clustering was done using the complete agglomeration method. Strong positive correlations are depicted by the orange color, strong negative correlations are depicted by the blue color. Different blocks are highlighted. The red block depicts unhealthy variables that strongly correlate with each other. The green block represents the strong correlation between different ASVs of *Prevotella* and *Intestinimonas* and its positive correlation with the rate of glucose disappearance and a negative correlation with insulin levels. Furthermore, a strong negative correlation between ASVs of *Bacteroides* and *Prevotella* is depicted (brown block).

**Figure S16.** Exploration of neighboring CpGs of cg04751533 (corresponding to the AFAP-1 gene) found in the model. (A) Spider plot depicting the neighboring CpGs of cg04751533 that differentiate between changes upon the autologous (red) versus changes upon allogenic (blue). (B) Spider plot depicting the neighboring CpGs of cg04751533 that differentiate between the non-responders (red) versus the responders (blue) at baseline.

**Figure S17.** UpSet plot depicting the number of subjects per panel and the number of subjects overlapping in different panels.
