# Supplementary material for: Effects of fecal microbiota transplant on DNA methylation in subjects with metabolic syndrome
Source: Gut Microbes. 2021 Nov 7;13(1):1993513. doi: 10.1080/19490976.2021.1993513 (PMC8583152; doi:10.1080/19490976.2021.1993513)
Supplement: Supplemental Material [file KGMI_A_1993513_SM2105.zip › Supplemental table captions.docx]

# Supplemental Information – Tables

**Table S1.** Characteristics of study subjects for the microbe-, metabolite-, and epigenetics panels.

**Table S2.** Sequences of the top 10 ASVs found in the model.

**Table S3**. PMBC cell fractions.

**Table S4.** Inter-marker correlations, Spearman’s rho.
