# Supplementary figures and images for: Effects of fecal microbiota transplant on DNA methylation in subjects with metabolic syndrome
Source: Gut Microbes. 2021 Nov 7;13(1):1993513. doi: 10.1080/19490976.2021.1993513 (PMC8583152; doi:10.1080/19490976.2021.1993513)

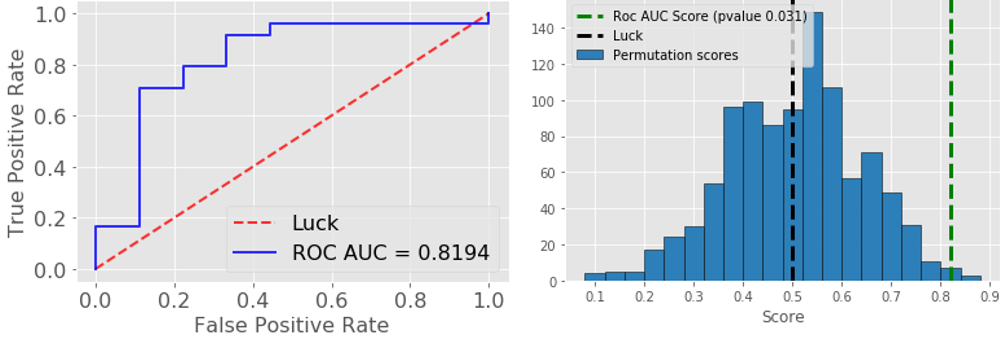

Supplement: Supplemental Material [file KGMI_A_1993513_SM2105.zip › Figure S1 and S2.png]

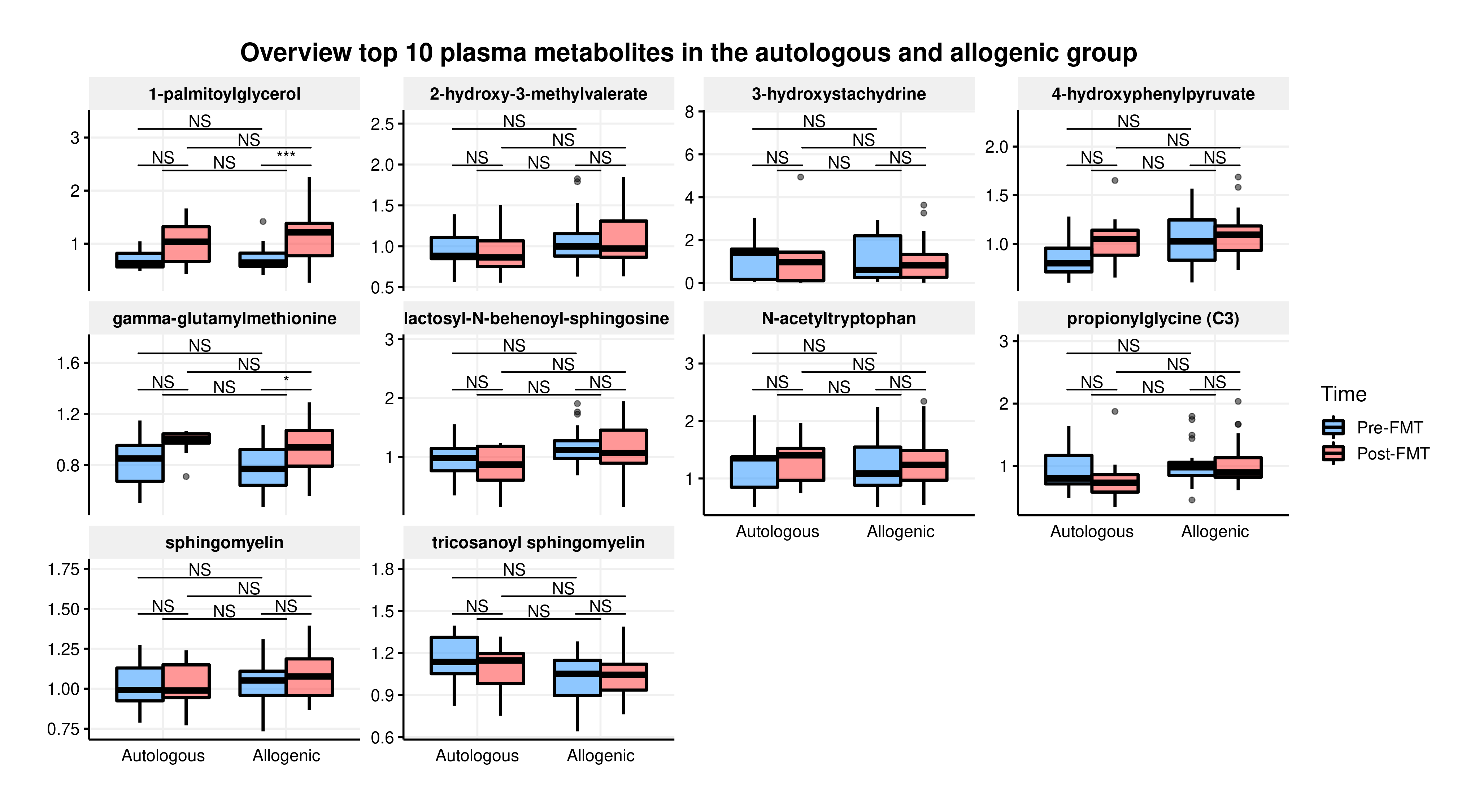

Supplement: Supplemental Material [file KGMI_A_1993513_SM2105.zip › Figure S10.png]

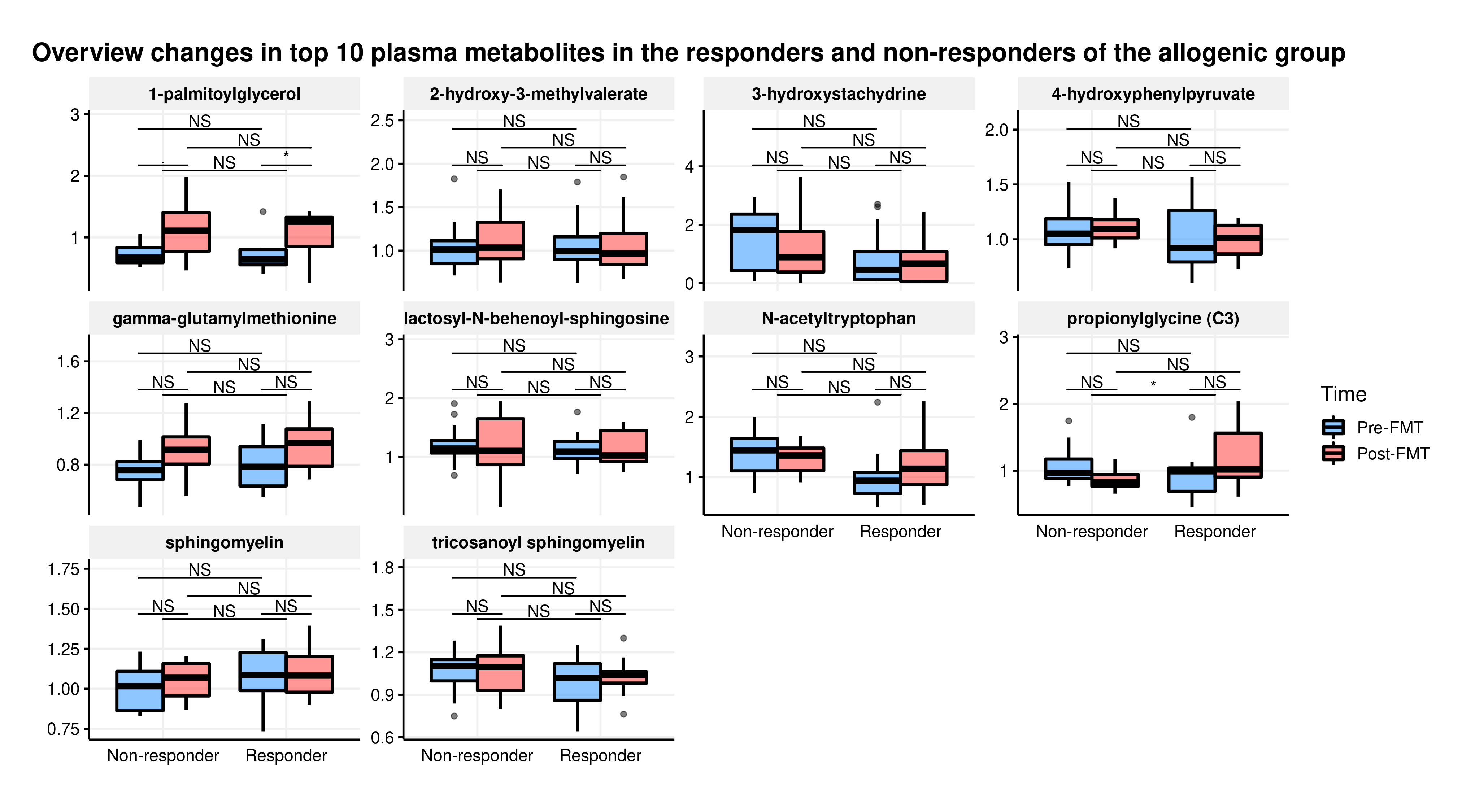

Supplement: Supplemental Material [file KGMI_A_1993513_SM2105.zip › Figure S11.png]

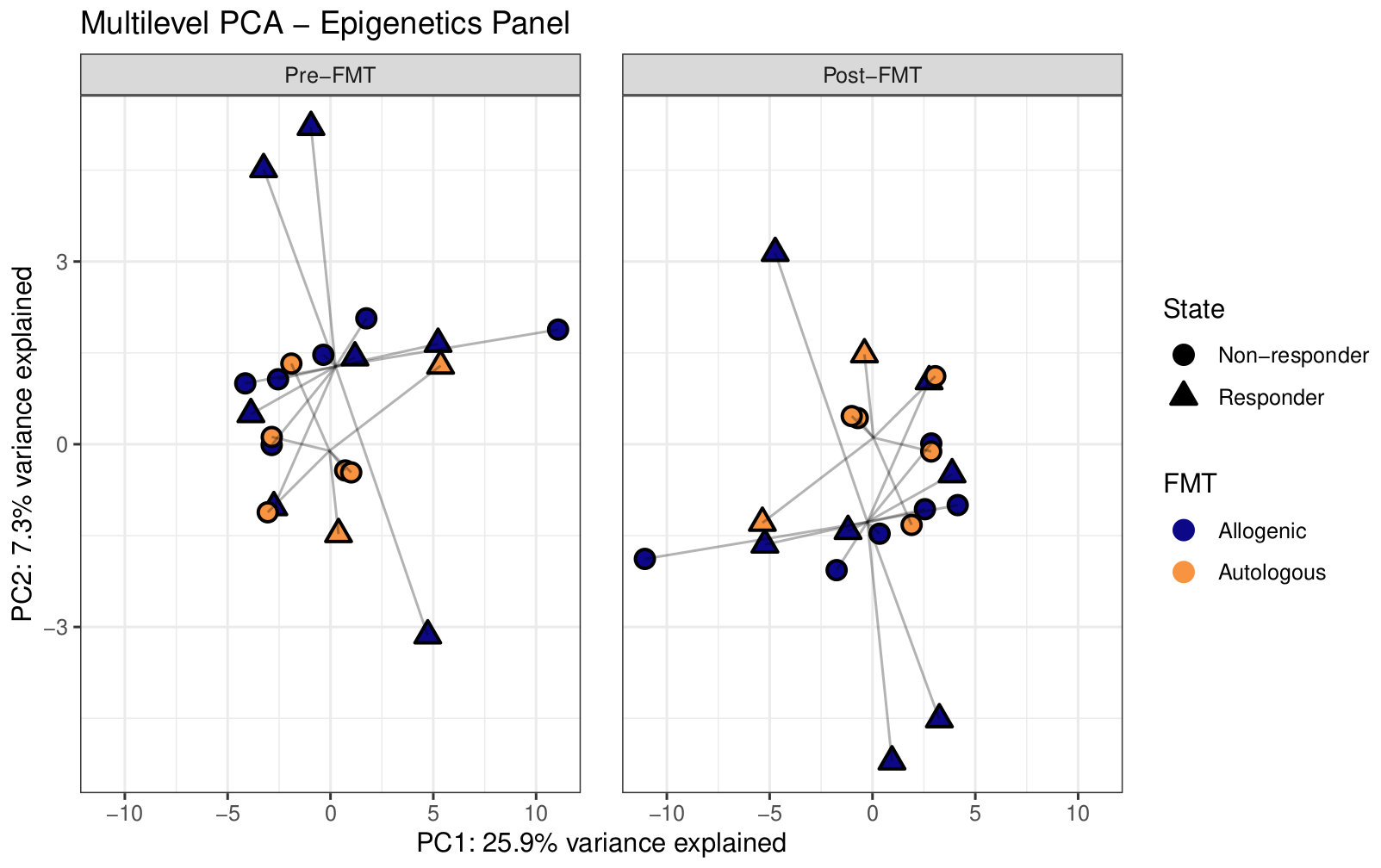

Supplement: Supplemental Material [file KGMI_A_1993513_SM2105.zip › Figure S12.png]

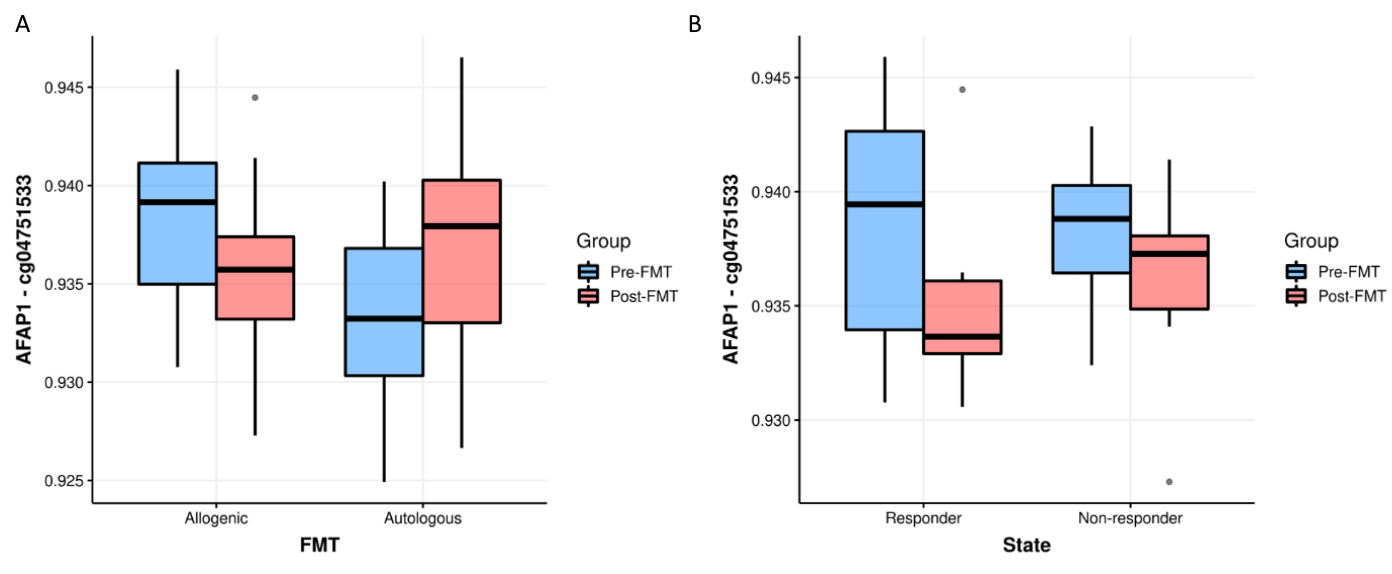

Supplement: Supplemental Material [file KGMI_A_1993513_SM2105.zip › Figure S13.png]

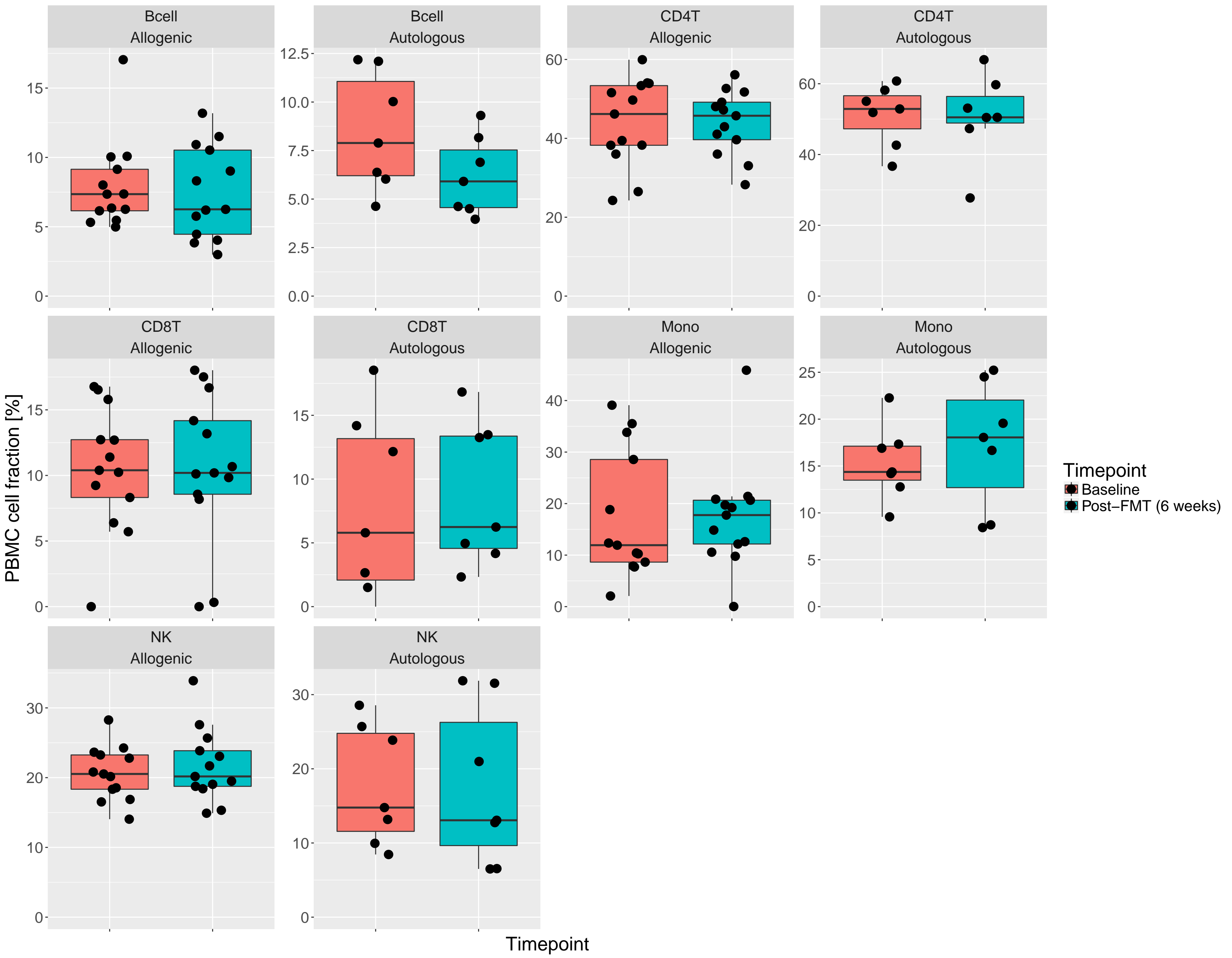

Supplement: Supplemental Material [file KGMI_A_1993513_SM2105.zip › Figure S14.pdf]

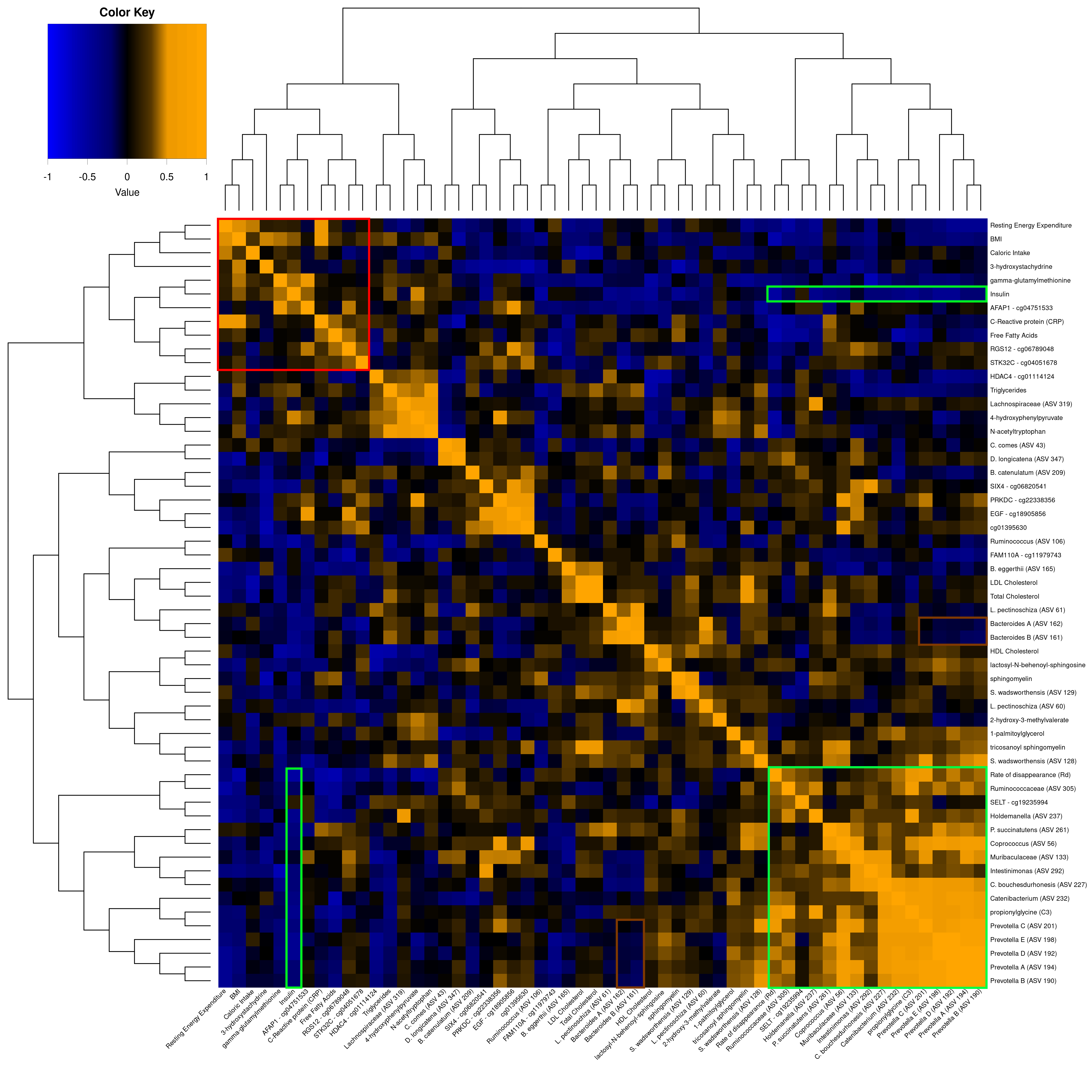

Supplement: Supplemental Material [file KGMI_A_1993513_SM2105.zip › Figure S15.png]

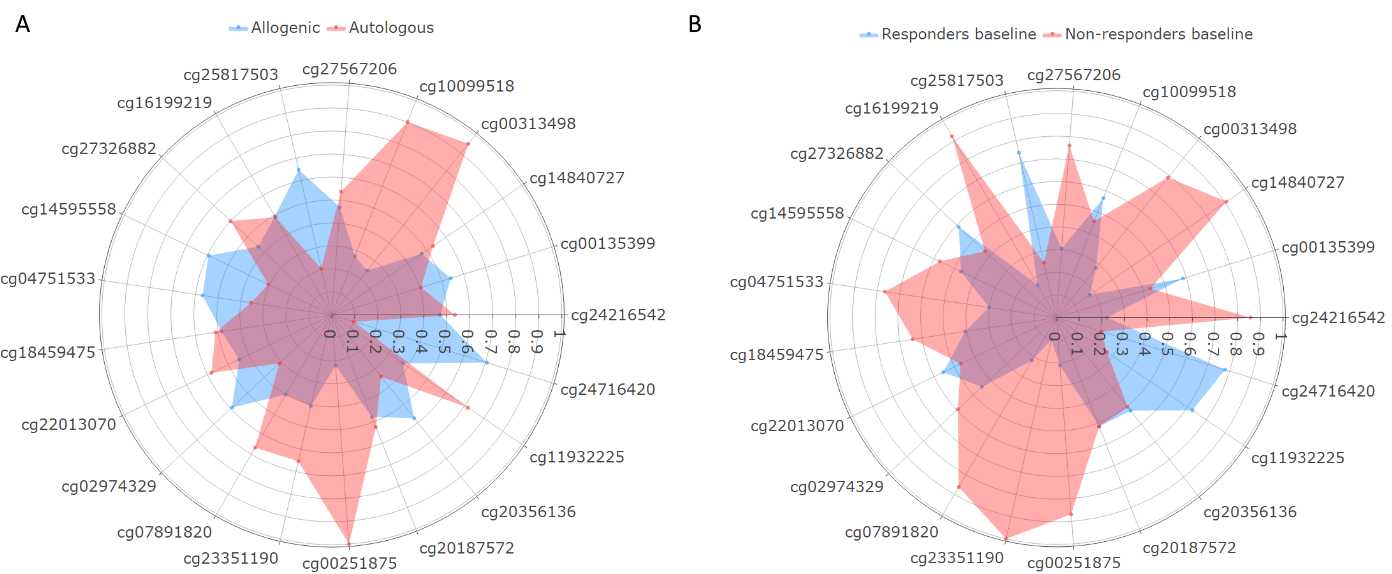

Supplement: Supplemental Material [file KGMI_A_1993513_SM2105.zip › Figure S16.png]

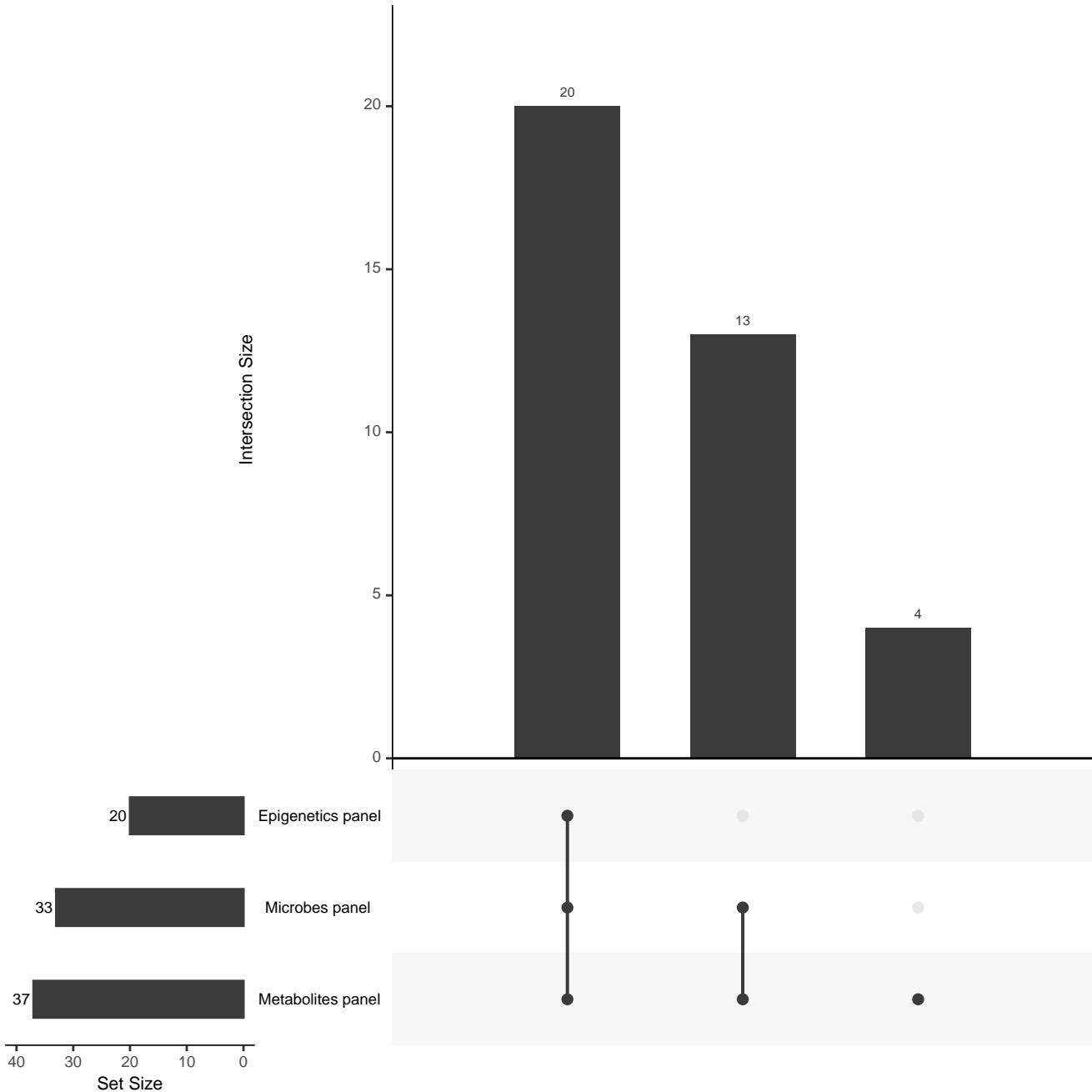

Supplement: Supplemental Material [file KGMI_A_1993513_SM2105.zip › Figure S17.pdf]

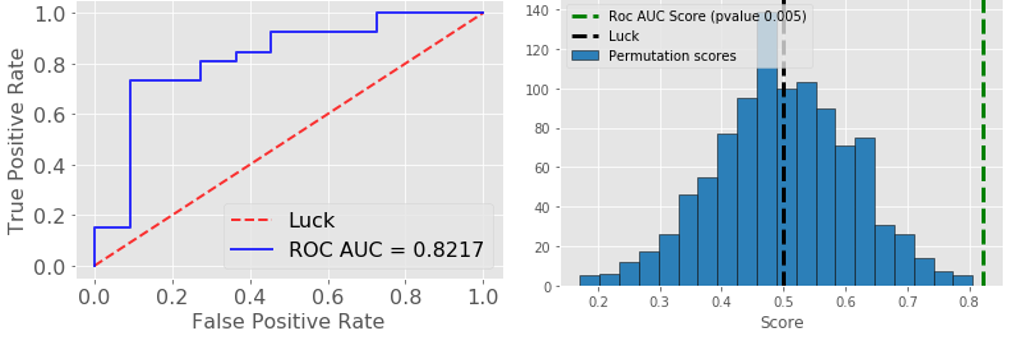

Supplement: Supplemental Material [file KGMI_A_1993513_SM2105.zip › Figure S3 and S4.png]

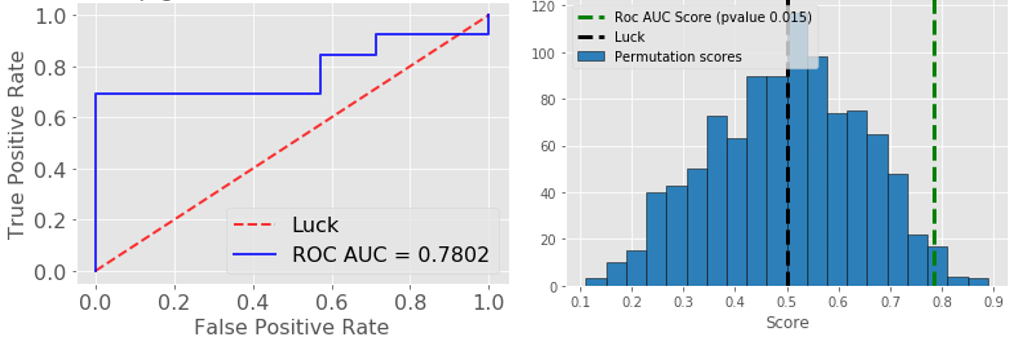

Supplement: Supplemental Material [file KGMI_A_1993513_SM2105.zip › Figure S5 and S6.png]

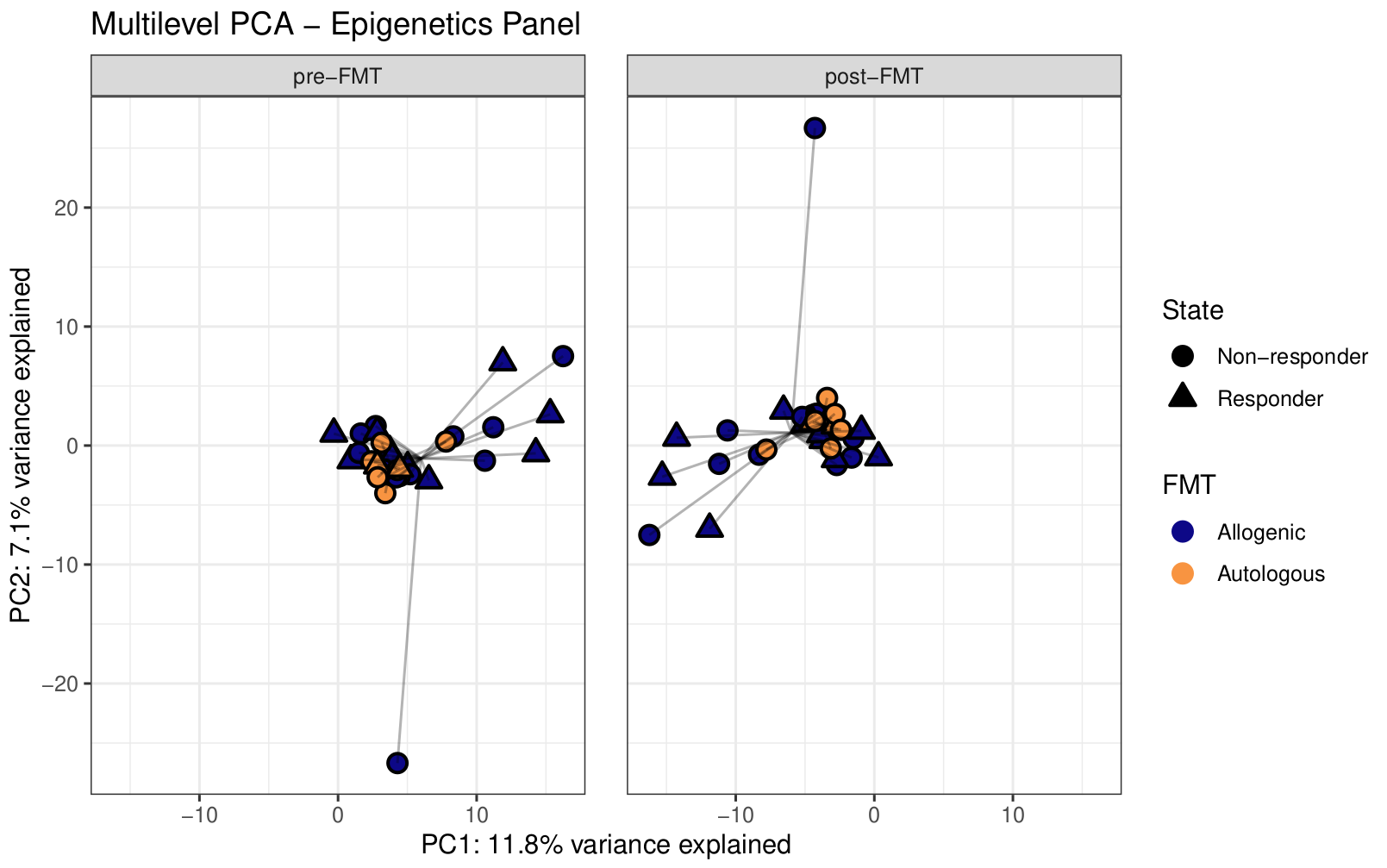

Supplement: Supplemental Material [file KGMI_A_1993513_SM2105.zip › Figure S7.png]

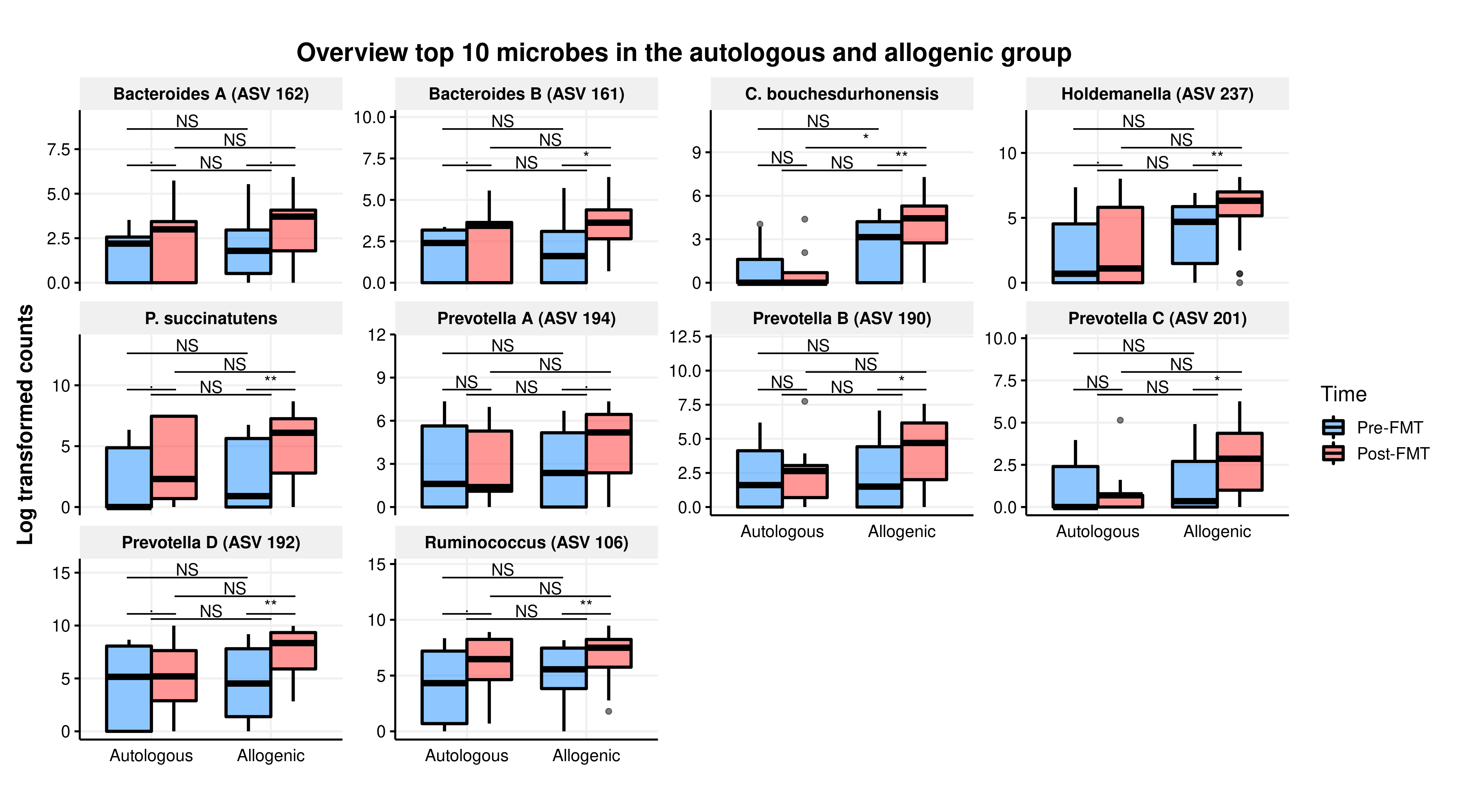

Supplement: Supplemental Material [file KGMI_A_1993513_SM2105.zip › Figure S8.png]

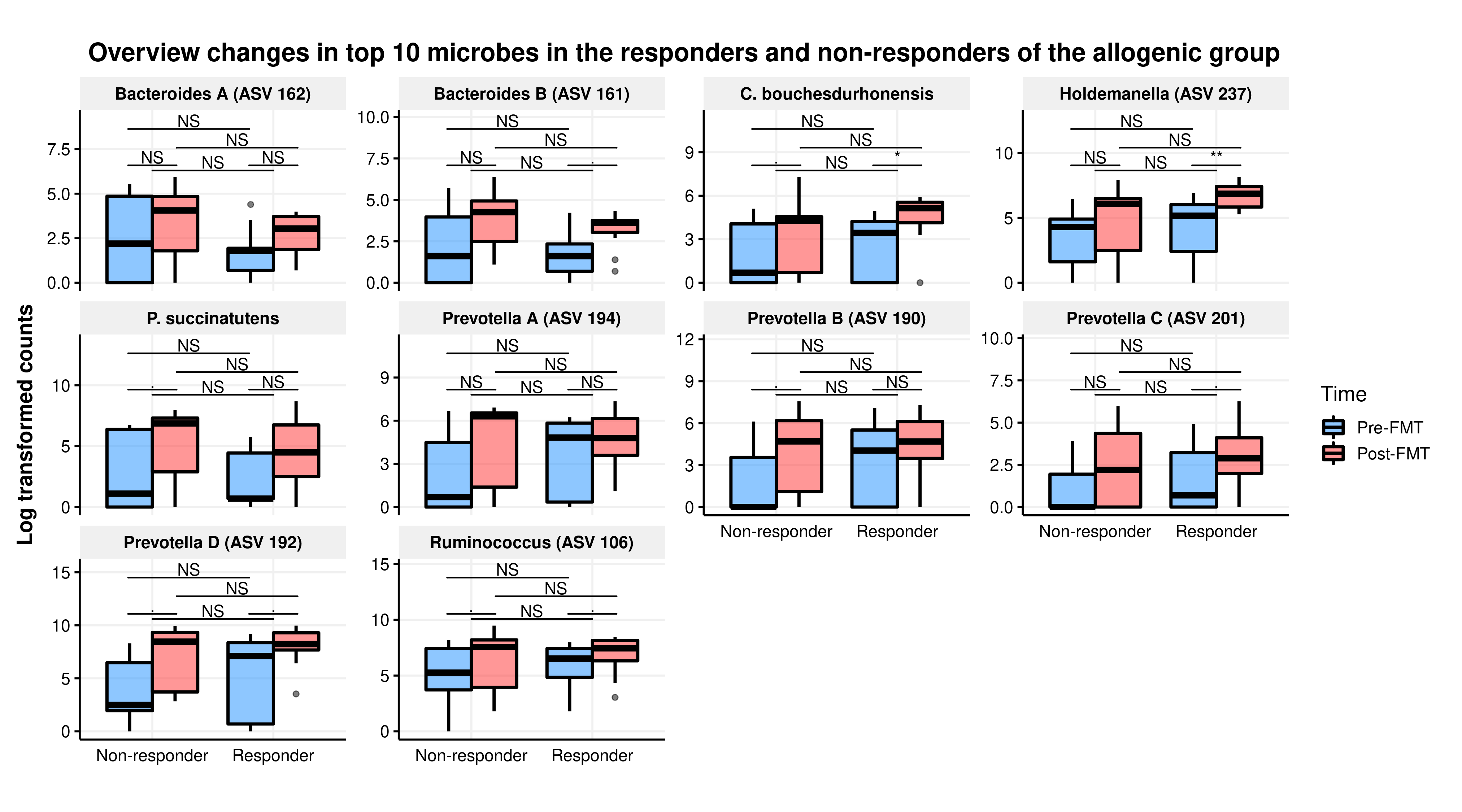

Supplement: Supplemental Material [file KGMI_A_1993513_SM2105.zip › Figure S9.png]
